# Effect of let-7a overexpression on the differentiation of conjunctiva mesenchymal stem cells into photoreceptor-like cells

**DOI:** 10.22038/ijbms.2019.32736.7859

**Published:** 2019-08

**Authors:** Fatemeh Ranjbarnejad, Samad Nadri, Alireza Biglari, Samira Mohammadi-Yeganeh, Mahdi Paryan

**Affiliations:** 1 Department of Genetics and Molecular Medicine, Zanjan University of Medical Sciences, End of Mahdavi Blvd, Shahrak-e Karmandan, 4513956111, Zanjan, Iran; 2 Department of Medical Nanotechnology, Zanjan University of Medical Sciences, End of Mahdavi Blvd, Shahrak-e Karmandan, 4513956111, Zanjan, Iran; 3 Cancer Gene Therapy Research Center, Zanjan University of Medical Sciences, End of Mahdavi Blvd, Shahrak-e Karmandan, 4513956111, Zanjan, Iran; 4 Department of Biotechnology, Shahid Beheshti University of Medical Sciences, Velenjak, 7th Floor, Bldg No 2 SBUMS, Arabi Ave, 19839-63113, Tehran, Iran; 5 Department of Research and Development, Production and Research Complex, Pasteur Institute, No 69, Pasteur Ave, 1316943551, Tehran, Iran

**Keywords:** Conjunctiva, Let -7a, Mesenchymal stem cells MiRNAs, Photoreceptor differentiation

## Abstract

**Objective(s):**

MicroRNAs (miRNAs) could regulate many cellular processes such as proliferation and differentiation. let-7a miRNA is one of the key regulators in the developmental transition of retinal progenitor cells into differentiated cells. Current evidence suggests that mesenchymal stem cells (MSCs) can isolate from various tissues such as bone marrow and conjunctiva. In this study, we investigated the effect of let-7a overexpression on induced differentiation of conjunctiva mesenchymal stem cells (CJMSCs) into photoreceptor-like cells.

**Materials and Methods:**

After isolation and characterization, CJMSCs were transduced with lentiviruses containing let-7a or empty vector. The effect of let-7a overexpression on expression of photoreceptor-specific markers was evaluated by quantitative real-time PCR (RT-qPCR) after 28 and 42 days of transduction.

**Results:**

The relative expression of rhodopsin and recoverin genes was evaluated by RT-qPCR in let-7a overexpressing cells, control vector transduced cells and untransduced CJMSCs (control cells). Our results indicated that following overexpression of let-7a, after 28 and 42 days of transduction, significant up-regulation in the expression of recoverin (574.7 and 43.9 folds) and rhodopsin (3334.7 and 53.1 folds) were observed, respectively.

**Conclusion:**

Our findings indicate that overexpression of let-7a microRNA can increase the expression of photoreceptor-specific genes in CJMSCs. Moreover, it is prospective that let-7a overexpression can use as an alternative protocol for the differentiation of mesenchymal stem cells into photoreceptors. It seems that the effect of let-7a on the differentiation of CJMSCs into photoreceptors is also time-dependent.

## Introduction

MicroRNAs (miRNAs) as short non-coding RNAs that are able to regulate gene expression and many cellular processes such as proliferation and differentiation (1, 2) by controlling transcription regulators (3).

**Table 1:** Specific primers used for qPCR amplification

**Name**	**Sequence (** **5'**→**3')**	**Amplicon length**	**Reference**
***TBP***	F: 5' GTT AGA AGG CCT TGT GCT CAC CCA CC 3' R: 5' AGA GCC ATT ACG TCG TCT TCC TGA ATC CC 3'	221 bp	[29]
***Recoverin***	F:5' GCC TTC TCC CTC TAC GAC 3' R: 5' CAT CTG TGG AGG GTC TTG G 3'	198 bp	[29]
***Rhodopsin***	F: 5' GGC TGG TCC AGG TAC ATC C 3' R: 5' GCC TCC TTG ACG GTG AAG 3'	179 bp	[29]
***Let-7a***	F: 5´ GGC TGA GGT AGT AGG TTG TAT AG 3´ R:5´ GAGCAGGGTCCGAGGT 3´	89 bp	[40]
***SNORD47***	F:5´ATC ACT GTA AAA CCG TTC CA 3´ R:5´ GAGCAGGGTCCGAGGT 3´	70 bp	[40]

**Figure 1:**
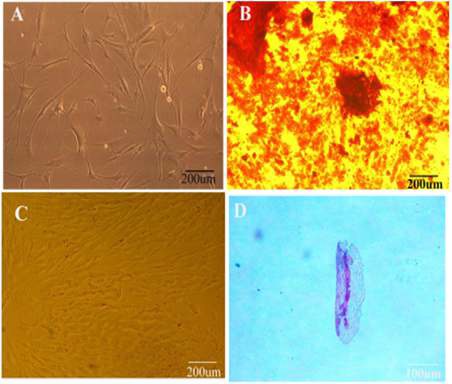
Morphological characteristics and *in vitro* differentiation of conjunctiva stromal fibroblast-like cells into mesenchymal lineages. (A) Conjunctiva stromal cells (passage 2) have differentiated into (B) mineralizing cells stained with alizarin red. (C) Adipocytes stained with Oil red O. (D) Chondrocytic lineage stained with alcian blue. (Magnification= ×20)

**Figure 2:**
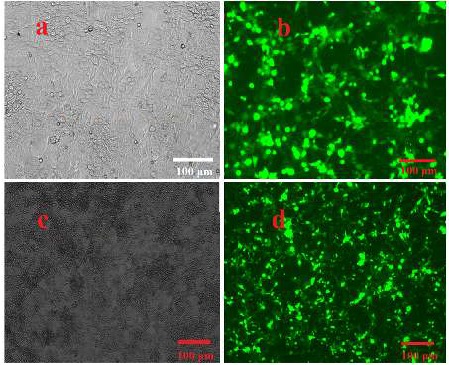
let-7a expression in HEK 293T cells. (A) Fluorescence and phase-contrast invert microscopy images of transduction efficiency of the let-7a overexpression vector after 24 hr (A, B) and 72 hr (C, D) of transduction (×40)

**Figure 3:**
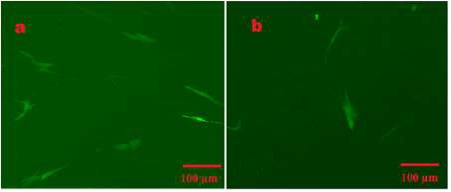
Fluorescence microscopy images of transduction efficiency of the let-7a overexpression vector (a) and empty vector (b) in CJMSCs after transduction, respectively (×80)

**Figure 4:**
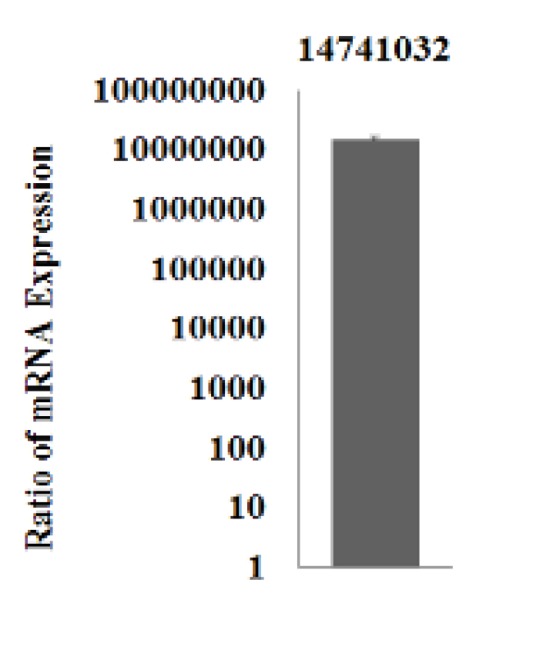
let-7a expression in CJMSCs transduced with let-7a. Expression level of let-7a assessed by qPCR in CJMSCs transduced with let-7a in comparison with empty vector transduced group on day 28. Data were normalized to the level of SNORD47 in each sample. Data are the mean ± standard deviation of three experiments

**Figure 5:**
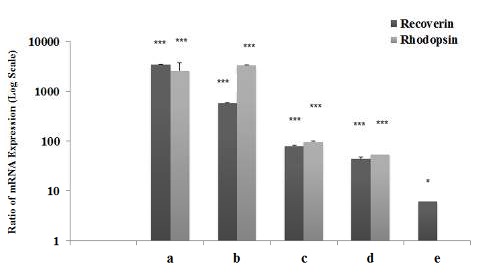
Expression levels of rhodopsin and recoverin assessed by real time RT-PCR in CJMSCs transduced with let7-a in comparison with the untransduced group (negative control) on day 28 (a), day 42 (c) and in comparison with empty-vector transduced group on day 28 (b) and day 42 (d), respectively. Expression levels of recoverin assessed by real time RT-PCR in empty-vector transduced CJMSCs in comparison with the untransduced group on day 28 (e). Asterisks indicate significant results (*P<0.05, ***P<0.0001)

Several studies have shown that the differentiation of a particular type of cells is regulated by specific miRNAs. For example, the role of miR-196a in osteogenic differentiation of MSCs (4), miR-150 in B cell differentiation (5), miR-1 in smooth muscle cell (6) and cluster miR-106b,25 in neuronal differentiation has been reported (7).

MicroRNA let-7a has the highest expression in neural stem cells (NSCs) (8) which promote neuronal differentiation by targeting Lin28A and Lin28B (9, 10) and plays important roles in maintaining the self-renewal potential of NSCs (11, 12). Moreover, let-7 suppresses the proliferation of NSCs and promotes neural and glial differentiation (7, 13, 14). It has been demonstrated that more than 250 miRNAs (15) have essential roles in the development of the mammalian retina (16). let-7 miRNA plays key roles in the regulation of mouse retina development. It has been shown that expression of let-7a increases over the period of retinal neurogenesis. Increased expression of miR-125, let-7, and miR-9 can accelerate the normal development in the retina (17).

The purpose of this study was to differentiate conjunctiva mesenchymal stem cells (CJMSCs) into photoreceptor-like cells by using let-7a overexpression *in vitro*. To assess differentiation, the expression of photoreceptor-specific genes was assessed in the let-7a-overexpressing CJMSCs.

## Materials and Methods

***Isolation of CJMSCs and differentiation of these cells into mesenchymal lineages***

Isolation of CJMSCs was performed according to a protocol modified by Nadri and Yazdani (18). In brief, 2–3 mm^2^ of conjunctiva biopsies were treated with supplemented hormonal epithelial medium (SHEM) including 50 mg/ml dispase II (Sigma Chemical Co). Epithelial sheets were separated and the isolated stromal tissue sections were cultured in DMEM/F-12 (1:1) (Gibco, USA) containing 10% knockout serum (Gibco, USA), 4 ng/ml basic-FGF (PeproTech, USA), 5 mg/ml insulin (Sigma-Aldrich, USA), and 10 ng/ml human LIF (Chemicon, Temecula, CA) and were incubated at 37 ^°^C with 5 % CO_2_ in a humidified chamber. 

To confirm mesenchymal characteristics of the isolated cells, the cells were treated with osteogenic (DMEM containing 50 μg/ml ascorbic acid 2-phosphate (Sigma-Aldrich, USA), 10 nM dexamethasone (Sigma-Aldrich, USA), and 10 mM b-glycerophosphate (Sigma-Aldrich, USA)), adipogenic (DMEM, including 50μg/ml indomethacin (Sigma-Aldrich, USA) and 100 nM dexamethasone (Sigma-Aldrich, USA)) and chondrogenic (DMEM containing 10 ng/ml transforming growth factor-beta (TGF-beta3; Sigma-Aldrich, USA), 500 ng/ml bone morphogenetic protein-6 (BMP-6), 10^-7^ M Dexamethasone (Sigma-Aldrich, USA), and 50 μg/ml ascorbate-2-phosphate (Sigma-Aldrich, USA), 50 μg/ml insulin-transferrin-selenium (ITS; Gibco, USA)) media for 21 days . In the next step, Alizarin red, Oil red, and Alcian blue staining were carried out, respectively for osteogenic, adipogenic and chondrogenic differentiation.

***Virus packaging and condensation***

The expression vector (pLenti-III-hsa-let7a-GFP Vector) which was purchased from the Abmgood Company (Canada), was kindly provided by Stem Cell Technology Research Center (Tehran, Iran). psPAX2 plasmid consisting of the gag/pol packaging genes and pMDG.2 plasmid including VSV-G were co-transduced with pLenti-III-hsa-let7a-GFP plasmid containing pri-let-7a (or pLenti-III-mir-GFP empty vector (control vector)) into HEK-293T cell line according to the manufacturer’s instructions (Open Biosystems, Huntsville, AL, USA). The media was replaced with the fresh one on the next day and supernatants containing lentiviruses were collected every 24 hrs for 3 days. The collected supernatants were kept at 4 ^°^C and viral concentration was performed by centrifuging at 40000xg for 2.5 hrs at 4 ^°^C. The concentrated viruses were aliquoted and freshly used for cell transduction whilst the rest was preserved at -70 ^°^C.

***Treatment of CJMSCs with lentiviruses containing pri-let-7a sequence***

Transduction of the cells with viruses was performed 3 times with a 2-day interval for recovery with multiplicity of infection (MOI) of 30. Briefly, the lentiviruses containing pri-let-7a sequence or pLenti-III-mir-GFP empty vector as the control were mixed with polybrene (4 μg/ml) (Sigma-Aldrich, USA) in serum-free media. The mixture was incubated at room temperature (RT) for 20 min and then added to the CJMSCs (passage 5). Since polybrene is fatal for the cells, the culture medium containing polybrene was replaced with fresh media after 6–8 hr. To positively select vector transduced CJMSCs, these cells were treated twice with 0.8 μg/ml and 1 μg/ml of puromycin (Thermo Fisher Scientific, USA) (19-21). The transduction process of GFP tagged-CJMSCs containing vector was investigated with fluorescence microscopy, and overexpression of let-7a was evaluated by qRT-PCR. let-7a-overexpressing cells, empty vector transduced cells, as well as untransduced cells (control cells) were incubated for 6 weeks to fallow differentiation into photoreceptor-like cells. 

***miRNA*** ***expression verification***

To assess the functionality of the constructs, overexpression of let-7a was evaluated by RT-qPCR in let-7a-overexpressing CJMSC compared with empty vector-transduced cells. After 28 days of transduction, total RNA extraction was performed using RNX™-Plus reagent (Cinnagen, Iran). cDNA synthesis was performed using stem-loop structure. The relative quantification of miRNAs was measured by RT-qPCR using specific primers for let-7a and SNORD47 as an internal control. All reactions were prepared in triplicate, and real-time PCR (ABI 7500, Applied Biosystem, Foster City, CA, USA) was performed as previously mentioned (22, 23).

***Gene expression***

Total RNA was extracted and reverse transcription was also performed and used for 40 cycles qPCR in real-time PCR (ABI 7500, Applied Biosystem, Foster City, CA, USA) with a total volume of 15 μl consisting of 6.5 μl of SYBR Green Master mix (Ampliqon, Denmark), 1 μl of cDNA, and 0.5 μl of each 500 nM gene-specific primer. real-time qPCR was performed using the following thermal cycling: 15 min at 95 ^°^C for initial enzyme activation, followed by 40 cycles of 15 sec at 95 ^°^C and 30 sec at 60–62 ^°^C. Ultimately, melting of PCR products was done at 50–90 ^°^C to confirm PCR specificity by melting curve analysis. The data were uniformly normalized to the TBP gene as the internal control. The sequences of primers are presented in Table 1. 

***Statistical analysis***

qPCR data were analyzed using comparative Ct (or 2-ΔΔCT) method introduced by Livak (24).The statistical significance of the differences between mean values of two groups was determined using two-independent-samples test (Mann-Withney; *P*<0.05).

## Results

***Cell culture and characterization of*** ***conjunctiva mesenchymal stem cells ***

The spindle-shaped mesenchymal stem cells derived from human conjunctiva stromal cells were cultured in DMEM media containing 10% FBS and were incubated at 37 ^°^C with 5% CO_2_ in a humidified chamber. To verify the mesenchymal nature of CJMSCs, the isolated cells were treated with appropriate osteo-, chondro-, and adipo induction media, and their differentiation was validated by staining methods containing Alizarin red (for osteogenic differentiation), Alcian blue (for chondrogenic differentiation), and Oil red (for adipogenic differentiation) (Figure 1).

***Production of vector transduced-HEK-293T cells***

psPAX2 and pMDG.2 plasmids were co-transduced with pLenti-III-GFP-hsa-let-7a (as well as empty vector) into the HEK-293T cell line. After 72 hrs, more than 90% GFP positive cells were observed (Figure 2)

***Induced differentiation of CJMSCs with overexpression of let-7a (expression of photoreceptor-specific markers)***

The transduction process of GFP tagged-CJMSCs containing vector was confirmed with fluorescence microscopy (Figure 3). Let-7a-overexpressing cells or empty vector containing cells were incubated for a long time (28 and 42 days) to initiate differentiation into photoreceptor-like cells. The overexpression of let-7a in vector-transduced cells was confirmed by qPCR (Figure 4).

The results of recoverin and rhodopsin genes expression in lentiviruses-transduced CJMSCs is presented in Figure 5**.** As shown in Figure 5, 28 days after vector transduction, q-PCR analysis demonstrated that expression of rhodopsin (3334.7 fold; *P≤*0.0001; 2574.4 fold; *P≤*0.0001) and recoverin (574.7 fold; *P≤*0.0001; 3468.3 fold; *P≤*0.0001) was significantly higher in let-7a-overexpressing CJMSCs compared with empty vector-transduced and untransduced CJMSCs, respectively. Furthermore, 42 days after vector transduction, data analysis of qPCR demonstrated that expression of rhodopsin (53.1 fold; *P≤*0.0001;96.3 fold; *P≤*0.0001) and recoverin (43.9 fold; *P≤*0.0001; 79.6 fold; *P≤*0.0001) was significantly higher in let-7a-overexpressing CJMSCs compared with empty vector transduced and untransduced CJMSCs, respectively. Also, data analysis of qPCR demonstrated that expression of recoverin (6 fold; *P≤*0.05) was significantly higher in empty vector transduced compared with untransduced CJMSCs (Figure 5). 

## Discussion

Mesenchymal stem cells (MSCs) have the potential to differentiate into adipose, bone, cartilage, tendon, and muscle tissue. To date, these cells have been derived from multiple tissue sources including periosteum (25), trabecular bone (26), adipose tissue (27), synovium (28), skeletal muscle (29), lung (30), deciduous teeth (31), as well as conjunctival tissue. These cells have a spindle-shaped morphology and are simply differentiated into osteo-adipo-chondro and neural cells (32, 33). The photoreceptor differentiation of a population of MSCs isolated from human eye conjunctiva has been performed by using an induction medium (32). 

In the current study, we focused on the effect of let-7a overexpression on photoreceptor differentiation of CJMSCs. Our results showed that overexpression of let-7a can induce the differentiation of CJMSCs into photoreceptor-like cells.

MSCs can differentiate into photoreceptor-like cells using a mixture containing Activin A, Taurin, and epidermal growth factors (34, 35). Previous studies have confirmed that merely 20–30% of CD90 positive MSCs induced by taurine, expressed photoreceptor-specific markers such as rhodopsin and recoverin (36, 37). miRNAs are one of the actual regulators of stem cell commitment and differentiation, both *in vivo* and *in vitro*. It has been shown that miR-125, let-7, and miR-9 increase during retinal development (17). 

CJMSCs which have spindle-shaped morphology and no expression of AE-5 (CK12), are different from conjunctival epithelial stem cells, which are relatively small and express cytokeratin 12 (CK12) (38). CJMSCs can differentiate into osteocyte, adipocyte, chondrocyte, neurocyte, and photoreceptor-like cells by using an appropriate induction medium (32, 39). More than 80% of these cells have a colony-formation capacity (32). Furthermore, CJMSCs retain these capacities for many passages (up to passage 10). Since isolated cells retain proliferation and differentiation potential for many passages, cost-effective MSC proliferation can be attained using this protocol without the necessity for specific growth factors and equipment.

In the present study, we studied the induced differentiation of CJMSCs into photoreceptor-like cells by using miRNAs only without any growth factors. The efficient role of let-7a in the fate of these cells was evaluated autonomously. 

By over-expressing of let-7a in CJMSCs, significant up-regulation of the rhodopsin gene was observed. Rhodopsin is expressed in rod photoreceptor cells and has a significant role in rod cell development (40). Therefore, we concluded that let-7a, as an inducer of rhodopsin expression in CJMSCs, could directly cause differentiation of CJMSCs into photoreceptor-like cells. In the present study, CJMSCs induced by let-7a, express photoreceptor-specific markers such as rhodopsin (a late-developing rod photoreceptor marker) and recoverin (an early developing photoreceptor marker) *in vitro* (36, 37). let-7a is one of the lethal-7 miRNA family members, which regulate the neuronal differentiation during brain development in the CNS (41). In the current study, let-7a overexpression triggered an increase in both rhodopsin and recoverin genes expression level. 

let-7a involves mainly NSC differentiation rather than proliferation (41). It could be confirmed by real-time qPCR for photoreceptor-specific markers. It is apparent that let-7a is involved in photoreceptor differentiation of CJMSCs. Therefore, it is prospective that let-7a overexpression can be used as an alternative to conventional differentiation approaches to photoreceptors.

In addition, this study provides evidence for the regulatory role of let-7a in CJMSCs proliferation. In the let-7a-overexpressing condition, cellular proliferation was delayed (data not shown).

In the present study, it has been observed a significant difference between the empty vector-transduced cells and untransduced CJMSCs (as negative control groups) in terms of gene expression. Similar findings have shown that the infection with the viral vector itself can cause strong variations in the gene-expression pattern of the host cell (42). In order to examine whether vector transduction or transgene expression effects the differentiation potential of CJMSCs, the expression level of rhodopsin and recoverin genes in empty vector-transduced cells was compared with untransduced cells (CJMSCs). In term of recoverin expression, there was a significant difference between the empty vector-transduced and untransduced CJMSCs. This result shows that transduction process has affected the differentiation potential of these cells. 

## Conclusion

In the current study, for the first time, it has been demonstrated that overexpression of let-7a microRNA induces differentiation of CJMSCs into photoreceptor-like cells. Our results indicated that let-7a-overexpressing CJMSCs expressed significantly specific photoreceptor genes in comparison with control cells.

Overall, the expression of photoreceptor-specific markers in let-7a-overexpressing CJMSCs can suggest a new approach for retinal differentiation and ongoing retinal regeneration applications.

## Source of Support

This work was supported by Deputy of Research and Technology, Zanjan University of Medical Science, Zanjan, Iran (grant no: A-10-892-7, ethics no: ZUMS.REC.1394.186). The results presented in this article were extracted from the MSc thesis of Ms. Fatemeh Ranjbarnejad.
